# Impact of Precision Medicine on Cancer Education in Japanese High Schools: A Questionnaire Survey

**DOI:** 10.1007/s13187-025-02697-w

**Published:** 2025-08-09

**Authors:** Hiromi Moriya, Yutaka Matsumoto, Yuko Ohnuki, Kazuyoshi Hosomichi

**Affiliations:** 1 Faculty of Nursing, Tokai University School of Medicine, Isehara, Kanagawa Japan; 2 Faculty of Medicine, Tokai University School of Medicine, Kanagawa, Japan; 3 School of Life Science, Tokyo University of Pharmacy and Life Sciences, Tokyo, Japan

**Keywords:** Precision medicine, Adolescents and young adults (AYAs), High school, Teacher perceptions, Genetic education

## Abstract

Despite being an umbrella concept that considers individual differences in genetics, environment, and lifestyle in cancer prevention, diagnosis, and treatment, a clear conceptual definition of precision medicine in cancer education is lacking in Japan. We conducted a questionnaire survey on the current status of cancer precision medicine and teachers’ readiness and challenges for its introduction. Of 2000 randomly selected Japanese high schools, 349 schools participated in the survey, from February to March 2024, through teachers in charge of cancer education. Quantitative and qualitative methods were used for analysis. Only 6% of the high schools implemented precision medicine education, which was rarer than education on causes and characteristics of adolescent and young adult (AYAs) cancers, familial and hereditary tumors, and cancer treatment in general. Quantitative analysis showed that teachers were generally positive about implementing precision medicine in cancer education, despite qualitative challenges such as lack of instructional skills, difficulties in providing attention to students, and lack of teaching time, student interest, or access to external resources. Despite its limited impact on cancer education in Japan, precision medicine can potentially play an increasingly important role in cancer biology, causation, prevention, screening, and education in AYAs. The study findings provide a rationale for incorporating precision medicine into cancer education in Japan.

## Introduction

Cancer is the most common cause of disease-related mortality among adolescents and young adults (AYAs) in high-income countries [1], and their cancer incidence and mortality could be reduced through equitable healthcare access, increased clinical trial registries, and early-stage cancer detection through symptom monitoring by patients and physicians [2]. Adolescents, especially those aged 15–19 years, have excellent opportunities to approach schooling-based population-level prevention. Cancer education for AYAs is limited worldwide, and considering cancer a disease of old age, most cancer-prevention education strategies address only lifestyle factors, such as smoking and alcohol use, and environmental factors, such as viruses [3–5]. In recent years, adolescents’ cancer education has included personalized cancer-risk reduction [6], which considers individual characteristics, such as genetic susceptibility. This is based on “Precision Medicine”—a new approach that considers individual differences in a person’s genes, environment, and lifestyle in disease prevention, diagnosis, and treatment [7]. In Japan, the limited human genetics literacy among young people necessitates a general introduction to genomic medicine that focuses on genetic factors in school education to distinguish it current environmental and lifestyle approaches to disease prevention [8]. To overcome fear and an avoidant perception of cancer, AYAs should undertake positive action to prevent cancer based on their individualized disease risk.

Since 2022, cancer education has been mandated in Japanese high schools [9], and the government has sequentially conducted cancer education training for high school teachers that aims to teach two aspects: self-health education (prevention of cancer in students) as well as human rights education (understanding and living with those undergoing cancer treatment) [4]. Thus, the current materials of the governmental cancer education model cover a range of topics, including a general overview of cancer, statistics, development, prevention, screening, treatment, palliative care, the psychology of survivors, and supporting survivors [10]. However, the model neither considers the genetic characteristics of carcinogenesis nor comprehensively acknowledges the existence of minority groups, such as AYAs with cancer, familial and hereditary tumors, and several idiopathic cancers. This nonrecognition of the differences in cancer risk and different properties of same-organ cancers that vary by the host human genome and genetic mutations/variants hinder effective cancer education. The non-incorporation of precision medicine into cancer education deprives an opportunity to educate on individual variations in biological cancer-risk factors, compounded by the lack of a knowledge base to understand pediatric-onset hereditary and familial tumors, with consequent erroneous disease-prevention behaviors whereby students with high familial cancer risk may enforce only lifestyle-based coping strategies.

Although promoting precision medicine in cancer education in Japan is important for cancer prevention among AYAs, the actual relationship between cancer education and precision medicine remains unelucidated. Therefore, we aimed to clarify the extent of precision medicine awareness in cancer education in upper secondary schools and ascertain how educators perceive its introduction and challenges.

## Methods

### Participants

Using stratified random selection among 5216 high schools with the Ministry of Education, Culture, Sports, Science, and Technology-assigned school codes in May 2023, we targeted 2000 schools, including 1,887 high schools, 69 higher vocational schools, 22 higher technical colleges, and 22 high schools. The survey respondents were the teachers in charge of cancer education, as recommended by the school principal (one per school). This observational study was conducted in compliance with the Declaration of Helsinki and the study protocol.

### Survey

The online survey, conducted from February 1 to March 31, 2024, used a Microsoft Form-based questionnaire that included three sections along with basic information. To determine the current status, 12 educational topics covered by national guidelines or educational practice were selected, with multiple-choice responses on whether each topic was addressed in current education or had training needs. Teachers’ attitudes included the original 17 items on the introduction of precision medicine; agreement with statements was rated using a 6-point Likert scale (0, not applicable; 1, strongly disagree; 2, disagree; 3, somewhat agree; 4, agree; 5, strongly agree). The survey items were selected based on existing research, including 5 items on awareness of dissemination [11] and 12 on readiness to use genomics in practice [12]. The summary included an open-ended statement regarding the harmful effects of cancer education.

### Data Analysis

Quantitative data are presented as frequencies and proportions. Data were compared using the chi-square test (two groups), and a two-sided significance level of 5% was considered statistically significant. Statistical analyses were performed using SPSS version 26.0 for Windows (SPSS, Inc., Tokyo, Japan). Content analysis was applied to the qualitative data. First, two researchers (HM and YM) intercepted the raw data to ensure only one meaning per sentence. Data were then categorized according to the commonality of meaning and heading, and two researchers (YO and KH) randomly sampled and evaluated 10% of the total data for content validity. A Scott’s agreement rate > 70% was considered to indicated reliable classification [13].

## Results

### Clinicodemographic Characteristics

Table 1 summarizes responses from 349 high schools across Japan (response rate: 17.5%). Regarding respondent attributes, the most common specialty and occupation were health and physical education (*n* = 252, 72.2%) and head teachers/teachers/school nurses (*n* = 310, 88.8%), respectively. The mean age and teaching experience of the respondents were 42.2 ± 10.7 and 18.0 ± 12.1 years, respectively; < 3% of respondents had a history of cancer, and only 9% and 34.4% had a national medical license and received cancer education training, respectively.

**Table 1:** Demographics and characteristics of the respondents

Demographics and characteristics		Frequency	Rate (%)	Mean ± SD
Age (years)				42.2 ± 10.7
Years of teaching experience (cumulative)				18.0 ± 12.1
Subject taught (specialization)	Health and physical education	252	72.2%	
	School nurse	66	18.9%	
	Science (biology)	9	2.6%	
	Other subjects	16	4.6%	
	No subject taught	6	1.7%	
Position	Principal/vice principal	11	3.2%	
	Supervising teacher/guidance teacher	17	4.9%	
	Head teacher/teacher/school nurse	310	88.8%	
	Lecturer from an external institution	11	3.2%	
History of cancer education training	Yes	120	34.4%	
	No	224	64.2%	
	No response	5	1.4%	
Personal history of cancer	Yes	9	2.6%	
	No	335	96.0%	
	No response	5	1.4%	
National medical license	Yes	31	8.9%	
	No	318	91.1%	

*SD*, standard deviation

### Cancer Education Program Content

Of the 12 survey items, no. 10, cancer precision medicine (21 schools, 6.0%); no. 11, cancer for AYAs (117 schools, 33.5%); and no. 12, familial and hereditary tumors (46 schools, 13.2%) had significantly lower coverage in the majority of the 349 high schools (Table 2).

**Table 2:** Content covered in cancer education programs

	Topic	Description	With education		Without education		*p*-value	
			Frequency	Rate (%)	Frequency	Rate (%)		
1	What is cancer?	Cancer is a group of diseases characterized by the uncontrolled growth and spread of abnormal cells. These cells can invade surrounding tissues and spread to distant sites	325	93.1%	24	6.9%	< 0.001	***
2	Cancer statistics	Cancer statistics provide data on the incidence, mortality, and survival rates of various types of cancer. This information is crucial for understanding cancer trends and developing effective prevention and treatment strategies	303	86.8%	46	13.2%	< 0.001	***
3	Cancer development	Cancer development is a complex process involving mutations, environmental factors, and lifestyle choices. It often progresses through multiple stages, from normal cells to precancerous changes to invasive cancer	257	73.6%	92	26.4%	< 0.001	***
4	Cancer prevention	Cancer prevention strategies aim to reduce the risk of developing cancer. These strategies may include lifestyle modifications and vaccinations	317	90.8%	32	9.2%	< 0.001	***
5	Cancer screening	Cancer screening involves using tests and procedures to detect cancer early, when it may be more treatable	264	75.6%	85	24.4%	< 0.001	***
6	Cancer treatment	Cancer treatment options vary depending on the type and stage of cancer. Common treatments include surgery, radiation therapy, and chemotherapy	202	57.9%	147	42.1%	0.003	**
7	Cancer palliative care	Cancer palliative care focuses on relieving symptoms and improving the quality of life for cancer patients and their families. It can be provided at any stage of cancer, from diagnosis to end-of-life care	199	57.0%	150	43.0%	0.009	**
8	The feelings of cancer patients	Cancer patients may experience a wide range of emotions, including fear, anxiety, sadness, anger, and isolation. It is important to provide emotional support and resources to help patients cope with these challenges	158	45.3%	191	54.7%	0.077	
9	A society living with cancer patients	Creating a supportive and inclusive society for cancer patients is essential. This includes raising awareness about cancer, reducing stigma, and providing resources for patients and their families	166	47.6%	183	52.4%	0.363	
10	Cancer precision medicine	This is based on “Precision Medicine”—a new approach that considers individual differences in a person’s genes, environment, and lifestyle in disease prevention, diagnosis, and treatment	21	6.0%	328	94.0%	< 0.001	***
11	Cancer in adolescents and young adults	Cancer in AYAs presents unique challenges due to the developmental stage and psychosocial needs of this population. It is important to provide specialized care and support for AYAs with cancer	117	33.5%	232	66.5%	< 0.001	***
12	Familial and hereditary tumors	Familial and hereditary tumors are cancers that occur due to inherited genetic mutations. Genetic testing and counseling may be recommended for individuals with a family history of cancer	46	13.2%	303	86.8%	< 0.001	***

Chi-square test. **p < 0.01, ***p < 0.001. *AYAs*, adolescents and young adults

Items 1 through 9 are the content designated as cancer education by the Japanese government; items 10 through 12 are the original items of this study

### Teachers’ Awareness and Readiness Regarding Precision Medicine

Intergroup comparison of the teachers’ responses, stratified by agreement (5, 4, and 3 points) and nonagreement (2, 1, and 0 points) groups, revealed significantly higher scores in the agreement group for all the five items regarding awareness of the promotion of precision medicine (Table 3). Among the 12 items on readiness for introduction, the agreement group scored significantly higher in all items, except for Readiness regarding precision medicine RE6, “I understand the role of the healthcare professional with respect to genetics;” RE8, “I can act with my peers to introduce precision medicine;” and RE9, “I can handle the topic of genetic testing,” which showed no significant between-group difference.

**Table 3:** Teachers’ awareness and readiness regarding precision medicine

	Teachers’ awareness and readiness regarding precision medicine	Agree		Not agree		*p*-value	
		Frequency	Rate (%)	Frequency	Rate (%)		
AW1	I better do precision medicine implementation	301	86.2%	48	13.8%	< 0.001	***
AW2	I am consistent with my values about precision medicine implementation	264	75.6%	85	24.4%	< 0.001	***
AW3	I do not find it difficult to implement precision medicine	198	56.7%	151	43.3%	0.012	*
AW4	I can add precision medicine to the current way of doing things	232	66.5%	117	33.5%	< 0.001	***
AW5	I think precision medicine implementation will be effective	276	79.1%	73	20.9%	< 0.001	***
RE1	I can decide about whether to implement precision medicine	210	60.2%	139	39.8%	< 0.001	***
RE2	I can consider the position of someone at high risk of developing cancer	279	79.9%	70	20.1%	< 0.001	***
RE3	I can be aware of the type of cancer	267	76.5%	82	23.5%	< 0.001	***
RE4	I can view cancer from the perspective of prevention, diagnosis, treatment, and prognosis prediction	288	82.5%	61	17.5%	< 0.001	***
RE5	I know how to obtain the latest knowledge on precision medicine	240	68.8%	109	31.2%	< 0.001	***
RE6	I understand the role of the healthcare professional with respect to genetics	185	53.0%	164	47.0%	0.261	
RE7	I can reflect on my prejudice and discrimination toward genetics	252	72.2%	97	27.8%	< 0.001	***
RE8	I can act with my peers to introduce precision medicine	187	53.6%	162	46.4%	0.181	
RE9	I can handle the topic of genetic testing	178	51.0%	171	49.0%	0.708	
RE10	I can consider the life perspectives of the easily affected	243	69.6%	106	30.4%	< 0.001	***
RE11	I can focus on the image of easy sufferers of cancer	237	67.9%	112	32.1%	< 0.001	***
RE12	I can take into consideration the cancer history of the student and the student’s family	231	66.2%	118	33.8%	< 0.001	***

*AW*, awareness regarding precision medicine; *RE*, readiness regarding precision medicine. Chi-square test. *p < 0.05, ***p < 0.001

### Difficulties and Challenges for Cancer Education

Among the 129 of 349 participants who responded to the free-form question, 117 responses were analyzed after excluding noncommittal responses, such as “none in particular” or “can’t think of anything,” When the smallest unit of problems in cancer education was a sentence that matched the research question, “What problems do teachers in charge of cancer education have?,” 264 units were extracted from 117 responses and, based on the commonality of meaning, were organized into categories A–E (Scott’s agreement rate: 84.2%), indicated in bold text, whereas codes are indicated with “”.

#### Demonstrating Learning Instruction Ability

The codes were related to the lack of a set in-school teaching plan for cancer education and the lack of necessary specialized knowledge among teachers, cancer education teaching materials, and motivation among some teachers imparting cancer education. There were problems with the formulation of teaching plans that matched the school’s characteristics, such as “We are not making progress in creating a form of cancer education that suits our school;” inability to come up with reasonable answers to students’ questions because of teachers’ lack of knowledge, such as “We cannot answer questions from students;” with preparing teaching materials, such as “There are no teaching materials that can be used;” and the inter-teacher dynamics, such as “It seems that the health and physical education teachers are not very interested.”

#### Considering Students Suffering from Cancer

The codes were related to the lack of a clear teaching method that considers those involved and the difficulty in understanding information about students and their families who have experienced cancer, such as “I cannot think of what kind of consideration I can give to my students” and “I worry while teaching whether the way I have been considered is correct” or “There is no way to find out that someone in their family has died of cancer.”

#### Allocating Appropriate Time to Cancer Education

The codes were related to insufficient time allocated to cancer-education classes and preparation for classes by teachers in charge of cancer education. Problems in securing time for students to study were as follows: “There are many things to teach besides health issues,” such as “career paths and preparation for qualification exams;” problems with securing time for teachers included: “It would be nice to request outside groups (medical professionals and patient groups) to manage and teach the class, but it takes time to make such an arrangement, so I can’t.”

#### Bringing Out Students’ Autonomy

The codes related to difficulty in gaining students’ interest and refraining from overstimulating students were identified based on problems related to students’ readiness, such as “Students think that cancer is an old person’s disease,” “Students get bored when specialized medical content is introduced,” and complicated teacher emotions, such as “The content is very delicate, so it is difficult to delve into the details.”

#### Making Effective Use of External Resources

Codes related to the inability to coordinate with lecturers because of lack of instructor dispatch system, insufficient funds for inviting lecturers, and lack of coordination with cancer-prevention systems in hospitals indicated problems related to barriers to the use of external resources, such as “in remote areas, there are not enough instructors to dispatch” and problems with fundraising, such as “we cannot pay the instructors’ fees or travel expenses.”

## Discussion

This investigation of the extent of precision medicine in high school cancer education showed how educators perceive its introduction and inherent challenges. Nationwide, individuals in charge of cancer education were mostly teachers who specialized in health and physical education, not in administrative positions, and had extensive contact with students. Thus, unlike online health education, educational effects through close communication were anticipated [14, 15]. Most of the teachers were in their early 40 s, had little teaching experience in cancer education, had never been diagnosed with cancer, and had insufficient knowledge about the subject. Most were possibly forced to tackle cancer education with lack of class preparation and life experience, whereby they could unintentionally provide medical advice that incurred a risk of legal and ethical issues despite policy guidelines issued by the school system. This differed from the situation in other countries, where cancer biology is covered in the curriculum and teachers who are already familiar with carcinogenic mechanisms and cancer-prevention methods undertake cancer education [16].

This study indicated that only 6% of high schools covered precision medicine, which was even less common than the education on causes and characteristics of AYA and familial and hereditary tumors, as well as cancer treatment. The most frequently covered content was cancer understanding and preventive behavior, especially cancer prevention, similar to that in other countries, where tobacco use, increased physical activity, weight control, dietary improvement, and protection from ultraviolet sun rays constitute cancer-preventive measures [17]. Despite being at the core of cancer education in Japan, the cancer-prevention content did not mention the need for individual risk-stratification based on knowledge of human genetics. Our findings corroborated the results of a previous study, which reported the paucity of education on genetic susceptibility, besides that by environmental factors, to cancer in school education [6], except for a small study which found that approximately 60% of teachers had experience in teaching genetics in cancer education in Japan [18]. This discrepancy is likely attributable to study size and population characteristics.

Incorporating the concept of genetic factors in personalized medicine into cancer-prevention education could potentially solve current issues with student autonomy in Japan. Dealing with cancers with onset at a young age can help students overcome the mistaken assumption that cancer or breast cancer prevention is only for older adults or women, respectively. For example, in the case of the *BRCA1* gene, the incidence of cancer increases rapidly with age from 31 to 40 years and is strongly associated with the presence and number of blood relatives with breast or ovarian cancer [19, 20], and the little-known male breast cancer [21]. However, this aspect is uncommonly covered in school education because cancer is still considered a complex disease caused by genetic and epigenetic changes [22], whereby the pace of cancer research may affect the selection of educational content.

Furthermore, our results suggest that the lack of clear cancer-prevention education methods for high-risk groups and families with cancer may be partly attributable to teacher, student, class time, and resource issues that add to other difficulties and challenges for cancer education. Students suffering from cancer present a fundamental problem in group education on cancer prevention, wherein balancing it with individualized attention to patients with cancer could detract from individualized cancer-prevention education. Teachers should focus on early-onset cancers, as these potentially affect AYAs. The cause of the cancer should be mentioned (e.g., thyroid cancer, malignant melanoma, testicular cancer, and Hodgkin lymphoma), and early signs and symptoms should be described. There is a need to clarify the approach to students with familial cancer and those at high risk of cancer. In the case of familial and hereditary tumors, cancer education has focused on families as a unit and has shown that students’ knowledge affects their families [23].

Long-term negative effects on school attendance, peer relationships, and employment among childhood and adolescent cancer survivors [24, 25] indicate the need for measures that avoid psychological aspects of the individual or discrimination or prejudice from those around them. Teachers’ attitudes toward the introduction of precision medicine for cancer were generally positive, which was similar to the responses of teachers in primary and secondary education who believed that it is necessary to teach genetics in cancer education [16].

## Conclusions

Precision medicine can potentially leverage an individual’s genetic information to facilitate more effective prevention strategies and early cancer detection as well as a framework for understanding future treatment options for AYAs. However, there is limited integration of precision medicine in Japan’s cancer-education curriculum for high school students. Teachers experienced challenges with precision medicine education, including a lack of instructional skills, difficulties in providing attention to students affected by cancer, and lack of teaching time, student interest, or access to external resources. Nevertheless, they expressed generally positive attitudes toward the introduction of precision medicine in cancer education. These findings provide a rationale for incorporating precision medicine into cancer education in Japan, wherein human genetics is not covered in high school curricula, and to thereby enable the next generation to acquire the latest medical knowledge to adopt a proactive approach to their health.

## Data Availability

The datasets generated and/or analyzed during this study are available from the corresponding author upon reasonable request.
